# Sex-dependent alteration of cardiac cytochrome P450 gene expression by doxorubicin in C57Bl/6 mice

**DOI:** 10.1186/s13293-016-0124-4

**Published:** 2017-01-07

**Authors:** Marianne K. O. Grant, Davis M. Seelig, Leslie C. Sharkey, Beshay N. Zordoky

**Affiliations:** 1 Department of Experimental and Clinical Pharmacology, University of Minnesota, 308 Harvard St S.E, Minneapolis, MN 55455 USA; 2 Veterinary Clinical Sciences Department, University of Minnesota, 1352 Boyd Ave, St. Paul, MN 55108 USA

**Keywords:** Doxorubicin, Cardiotoxicity, Sex, Cytochrome P450

## Abstract

**Background:**

There is inconclusive evidence about the role of sex as a risk factor for doxorubicin (DOX)-induced cardiotoxicity. Recent experimental studies have shown that adult female rats are protected against DOX-induced cardiotoxicity. However, the mechanisms of this sexual dimorphism are not fully elucidated. We have previously demonstrated that DOX alters the expression of several cytochrome P450 (CYP) enzymes in the hearts of male rats. Nevertheless, the sex-dependent effect of DOX on the expression of CYP enzymes is still not known. Therefore, in the present study, we determined the effect of acute DOX exposure on the expression of CYP genes in the hearts of both male and female C57Bl/6 mice.

**Methods:**

Acute DOX cardiotoxicity was induced by a single intraperitoneal injection of 20 mg/kg DOX in male and female adult C57Bl/6 mice. Cardiac function was assessed 5 days after DOX exposure by trans-thoracic echocardiography. Mice were euthanized 1 day or 6 days after DOX or saline injection. Thereafter, the hearts were harvested and weighed. Heart sections were evaluated for pathological lesions. Total RNA was extracted and expression of natriuretic peptides, inflammatory and apoptotic markers, and CYP genes was measured by real-time PCR.

**Results:**

Adult female C57Bl/6 mice were protected from acute DOX-induced cardiotoxicity as they show milder pathological lesions, less inflammation, and faster recovery from DOX-induced apoptosis and DOX-mediated inhibition of beta-type natriuretic peptide. Acute DOX exposure altered the gene expression of multiple CYP genes in a sex-dependent manner. In 24 h, DOX exposure caused male-specific induction of Cyp1b1 and female-specific induction of Cyp2c29 and Cyp2e1.

**Conclusions:**

Acute DOX exposure causes sex-dependent alteration of cardiac CYP gene expression. Since cardiac CYP enzymes metabolize several endogenous compounds to biologically active metabolites, sex-dependent alteration of CYP genes may play a role in the sexual dimorphism of acute DOX-induced cardiotoxicity.

**Electronic supplementary material:**

The online version of this article (doi:10.1186/s13293-016-0124-4) contains supplementary material, which is available to authorized users.

## Background

Doxorubicin (DOX) is a very effective chemotherapeutic agent commonly used to treat both hematological malignancies and solid tumors [1]. Nevertheless, the clinical use of this highly effective agent is limited by dose-dependent cardiotoxicity that can progress to cardiac dysfunction and heart failure [2]. The exact mechanism of DOX-induced cardiotoxicity is not fully elucidated; however, oxidative stress, mitochondrial dysfunction, derailed molecular signaling, inflammation, and apoptotic cell death are believed to be important players [3]. Since cardiac dysfunction does not occur in every DOX-treated cancer patient, there is a growing interest in identifying risk factors that predispose some patients to DOX-induced cardiotoxicity [4, 5]. Several factors have been shown to increase the risk of DOX-induced cardiotoxicity including total cumulative dose, pre-existing cardiovascular disease, and combination treatment with other cardiotoxic chemotherapeutic agents such as trastuzumab [5]. However, there is inconclusive and conflicting evidence for the role of sex as a risk factor for DOX-induced cardiotoxicity. It is widely accepted that female sex is a risk factor for DOX-induced cardiotoxicity in pediatric cancer patients [6, 7]; nevertheless, adult female patients have been shown to be less sensitive to DOX-induced cardiac adverse effects in some studies [1, 8, 9]. Female patients in this age group are likely to have high levels of sex hormones, suggesting a key role of sex hormones in mediating the protective effect of female sex against DOX-induced cardiotoxicity. In accordance with this notion, sex has not been shown to be a significant risk factor for DOX-induced cardiotoxicity in elderly cancer patients [10], implying a diminishing protective effect of female sex with the decline of sex hormone levels in the elderly. In more controlled experimental studies, we and others have shown that adult female rodents are protected against chronic DOX-induced cardiotoxicity [11–16]. Several mechanisms have been postulated to explain sexual dimorphism of chronic DOX-induced cardiotoxicity including differences in myocardial energy metabolism, mast cell activation, and cardiolipin remodeling [11, 12, 14]. Since the mechanisms of acute DOX-induced cardiotoxicity may be different from those of chronic cardiotoxicity [17], it is important to investigate the mechanisms of sex-related differences in acute DOX-induced cardiotoxicity.

We have demonstrated that acute DOX-induced cardiotoxicity alters the expression of several cytochrome P450 (CYP) enzymes in the H9c2 cardiomyoblasts in vitro [18] and in the hearts of male Sprague Dawley rats in vivo [19]. DOX-induced alteration of CYP enzymes may have important toxicological implications, due to the role of CYP enzymes in the metabolism of several biologically active endogenous compounds including arachidonic acid and sex steroids [20]. Sex-divergent expression of CYP genes has been well studied in the liver; however, a few studies have also reported sex-divergent expression of CYP genes in other organs [21]. Of interest, a recent study has demonstrated sex-dependent expression of some CYP genes in the rodent heart [22]. Nevertheless, the sex-dependent effect of DOX on CYP gene expression is still not known. Therefore, in the present study, we determined the effect of acute DOX exposure on the gene expression of CYP enzymes in the hearts of both male and female C57Bl/6 mice to determine the interaction between sex and DOX exposure in regulating CYP gene expression. We demonstrate for the first time that DOX-induced alteration of CYP gene expression is sex-dependent which may explain, at least in part, the sex-related differences of DOX-induced cardiotoxicity.

## Methods

### Animals

All experimental procedures involving animals have been approved by the Institutional Animal Care and Use Committee (IACUC) at the University of Minnesota. All animals were housed under environmentally controlled conditions in an Association for Assessment and Accreditation of Laboratory Animal Care (AAALAC) accredited facility according to the National Institutes of Health (NIH) guidelines and had free access to food and water during the study period. Twelve-week-old male (*n* = 31) and female (*n* = 26) C57Bl/6 mice were purchased from Charles River Laboratories (Raleigh, NC). After a 1 week acclimation period, mice were randomized to receive either 20 mg/kg DOX by intraperitoneal (IP) injection (DOX group) or equivalent volume of sterile normal saline (control group). Twenty-four hours after the DOX or saline injection, a cohort of mice (8 male-control, 8 male-DOX, 8 female-control, and 8 female-DOX) were humanely euthanized. The hearts were collected, washed in ice-cold phosphate buffered saline solution, flash frozen in liquid nitrogen, and stored at −80 °C until further analysis. Another cohort of mice (6 male-control, 9 male-DOX, 5 female-control, and 5 female-DOX) were followed up daily for 6 days. Mortality was observed only in the male-DOX group. By the 5th day after the DOX injection, 4 male mice had been found dead in their cages. On the 5th day, echocardiography was performed on the surviving mice (6 male-control, 5 male-DOX, 5 female-control, and 5 female-DOX). Another DOX-treated male mouse died just after the echocardiography. Although we performed daily checks on these mice to humanely euthanize any mouse showing marked morbidity, it seems that the mice’ health deteriorated rapidly within a 24-h period (between our daily checks). The surviving mice (6 male-control, 4 male-DOX, 5 female-control, and 5 female-DOX) were humanely euthanized and their hearts were collected, washed in ice-cold phosphate buffered saline, a section from the left ventricle was cut for histopathology analysis, and the remaining tissue was flash frozen in liquid nitrogen, and stored at −80 °C until further analysis.

### Echocardiography

On the fifth day following DOX administration, echocardiography was performed using a Vevo® 2100 system (VisualSonics, Inc., Toronto, Ontario, Canada). Mice were anesthetized in a chamber with 5% isoflurane and 100% oxygen, and anesthesia was maintained with 1–1.5% isoflurane while the mouse was securely placed in a supine position for the procedure. The total time for each procedure was approximately 10–20 min. The following measures were obtained: diastolic and systolic areas and their volumes, stroke volume, cardiac output, ejection fraction, fractional shortening, and left ventricular wall thickness.

### Histopathology

Tissue sections were collected at the same level of the left ventricle, fixed in 10% neutral buffered formalin, processed, and embedded in paraffin using standard methods. Thereafter, four-micron tissue sections were cut and stained with hematoxylin and eosin (HE) or trichome stain. Histopathologic evaluation was performed by a board certified veterinary pathologist who was blinded to the sex and the received treatment. The cardiac lesions were analyzed using a modified version of a myocardial biopsy scoring system [23]. Each stained section was examined for (a) myocyte vacuolization, (b) myocyte necrosis, (c) myocyte loss, and (d) interstitial cellularity (e.g., fibrosis or inflammation). For myocyte vacuolization, sections were principally scored based upon the number of cardiomyocytes with cytoplasmic vacuoles as follows: 0, no vacuolization observed; 1, few (<25%) cells with vacuoles; 2, occasional (26–50%) cells with vacuoles; 3, moderate numbers (51–75%) of cells with vacuoles; and 4, many cells (>76%) with vacuoles. For any section in which individual severely vacuolated cardiomyocytes (i.e., Adria cells) were identified, the score was elevated 1 point. For myocyte necrosis and loss, sections were scored based upon the number of affected cells as follows: 0, no necrosis or loss observed; 1, rare (1–5%) cells affected; 2, few (6–10%) cells affected; 3, modest number (11–15%) of cells affected; and 4, many (≥16%) cells affected. Interstitial cellularity, which comprised inflammation or fibrosis, was assessed as follows: 0, absent; 1, minimal interstitial infiltration; 2, mild interstitial infiltration; 3, moderate interstitial infiltration; and 4, marked interstitial infiltration.

### RNA extraction

Total RNA from the frozen tissues was isolated using TRIzol® reagent (Life Technologies, Carlsbad, CA) according to the manufacturer’s instructions, and quantified by measuring the absorbance at 260 nm using a Nanodrop 8000 spectrophotometer (Thermo Fisher Scientific, Wilmington, DE). The quality of the extracted RNA was determined by measuring the RNA integrity number (RIN) values (Additional file 1 shows a representative figure of RIN measurements) using an Agilent 2100 Bioanalyzer (Santa Clara, CA). Thereafter, first-strand cDNA was synthesized by using the High-Capacity cDNA reverse transcription kit (Life Technologies, Carlsbad, CA) according to the manufacturer’s instructions. In brief, 1.5 μg of total RNA from each sample was added to a mix of 1.0 μl MultiScribe™ reverse transcriptase (RT), 2.0 μl 10X RT buffer, 0.8 μl 25X dNTP mix (100 mM), 2.0 μl 10X RT random primers, and 4.2 μl nuclease-free water. The final reaction mix was kept at 25 °C for 10 min, heated to 37 °C for 120 min, heated for 85 °C for 5 min, and finally cooled to 4 °C in a Tetrad 2 thermal cycler (Bio-Rad Laboratories, Inc., Hercules, CA).

### Real-time PCR

Quantitative analysis of specific mRNA expression was performed by real time-polymerase chain reaction (PCR), by subjecting the resulting cDNA to PCR amplification using 384-well optical reaction plates in the ABI 7900HT instrument (Applied Biosystems, Foster City, CA). The 20-μl reaction mix contained 1 μl of cDNA sample, 10 μl of SYBR Green Universal Mastermix (Life Technologies, Carlsbad, CA), 0.025 μl of 30 μM forward primer and 0.025 μl of 30 μM reverse primer (40 nM final concentration of each primer), and 8.95 μl of nuclease-free water. We assessed acute DOX-induced cardiotoxicity by measuring the gene expression of atrial natriuretic peptide (ANP) and B-type natriuretic peptide (BNP) as markers of cardiac stress [19], cyclooxygenase-2 (COX-2) as an inflammatory marker [24], and Bcl-2 associated X protein/B-cell lymphoma-2 (BAX/Bcl-2) as an apoptotic marker [24]. Since we previously reported changes of certain CYP genes by acute DOX exposure in male rats [19], the mouse orthologs of those rat CYP genes were selected for the current study. We used β-actin as a house-keeping gene, and its expression was not significantly different between sexes or between treatments. To confirm that the observed changes were due to actual changes in the genes of interest, some analyses were repeated using 18S as a housekeeping gene, and similar results were obtained. The primers used in the current study were chosen from previously published studies [25–30], checked with the Primer-BLAST on-line tool [31], and are listed in Additional file 2. After sealing the plate with an optical adhesive cover, thermocycling was initiated at 95 °C for 10 min, followed by 40 PCR cycles of denaturation at 95 °C for 15 s, and annealing/extension at 60 °C for 1 min. Melting curve was performed by the end of each cycle to confirm the specificity of the primers and the purity of the final PCR product.

### Real time-PCR Data analysis

The analysis of real time-PCR data was performed using the relative gene expression i.e., (ΔΔCT) method as described in Applied Biosystems User Bulletin No.2 and explained further by Livak et al. [32]. In brief, the data in all groups are presented as the fold change in gene expression normalized to the endogenous reference gene (β-actin) and relative to male control animals of the same time point.

### Statistical analysis

Statistical analysis was performed using the GraphPad Prism software (version 7.01, 12 June 2016, La Jolla, CA). Results are presented as mean ± SEM. Comparisons among different sex and treatment groups were done by 2-way Analysis of Variance (ANOVA), followed by Tukey’s multiple comparison test as a post-hoc analysis. To correct for multiple comparisons, a false discovery rate of 5% was applied for *p* values of DOX effect, sex effect, and interaction effect on CYP gene expressions by the Two-stage linear step-up procedure of Benjamini, Krieger, and Yekutieli. Histopathologic grading of lesions is presented as median with 95% confidence interval of the median. Statistical analysis for histopathologic grading was performed using Kruskal-Wallis non-parametric test. *p* or *q* values of <0.05 were considered statistically significant.

## Results

### Female mice are less sensitive to DOX-induced cardiotoxicity than males

**Fig. 1:**
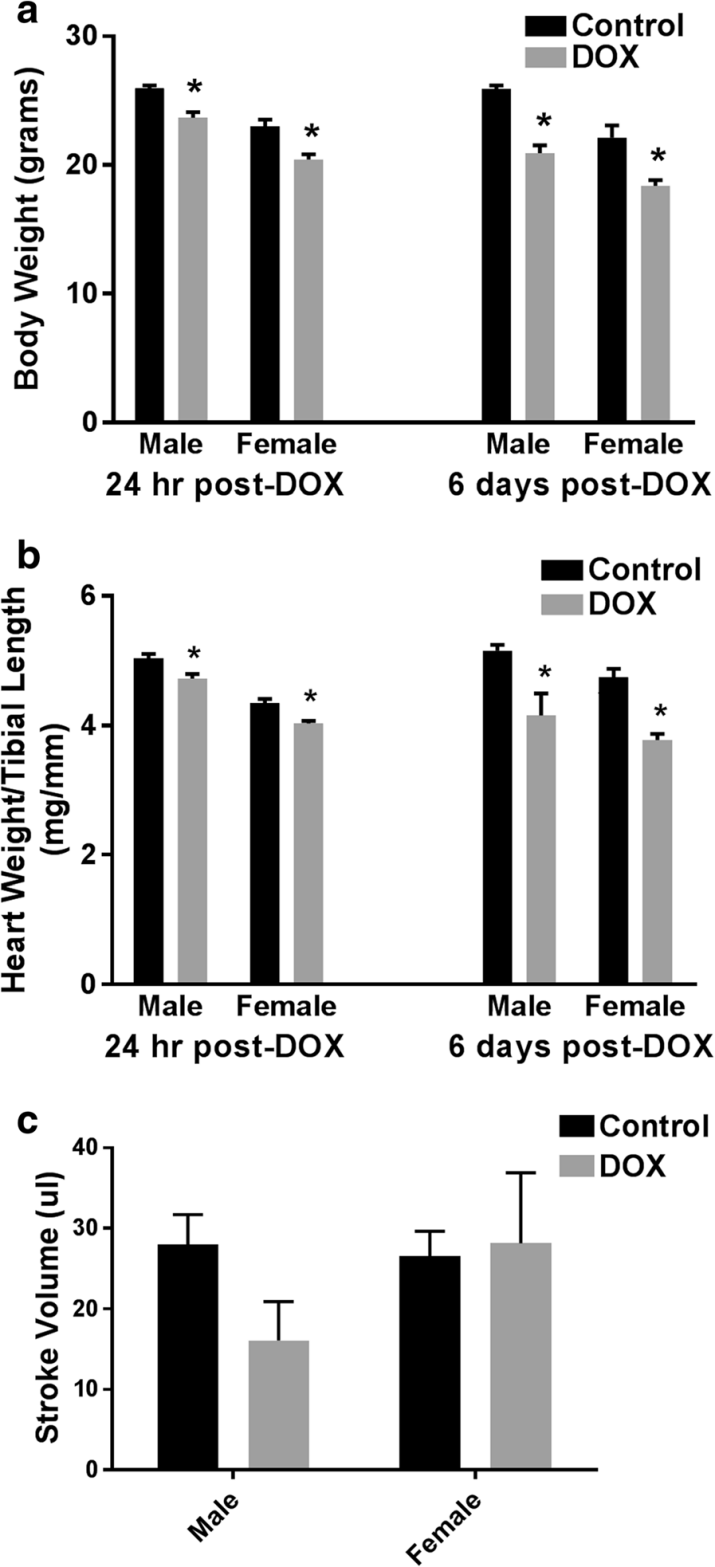
Adult male and female C57Bl/6 mice were administered a single intraperitoneal injection of 20 mg/kg DOX or equivalent volume of sterile saline. **a** Body weight and **b** heart weight to tibial length were measured 24 h (*n* = 8 per group) or 6 days (*n* = 4–6 per group) after the administration of DOX or saline in male or female mice. **c** Stroke volume was assessed by trans-thoracic echocardiography 5 days after DOX or saline administration in male or female mice (*n* = 5–6 per group). Values are expressed as mean ± SEM. *p < 0.05, compared to saline-treated mice of the same sex by Tukey’s post-hoc analysis

**Fig. 2:**
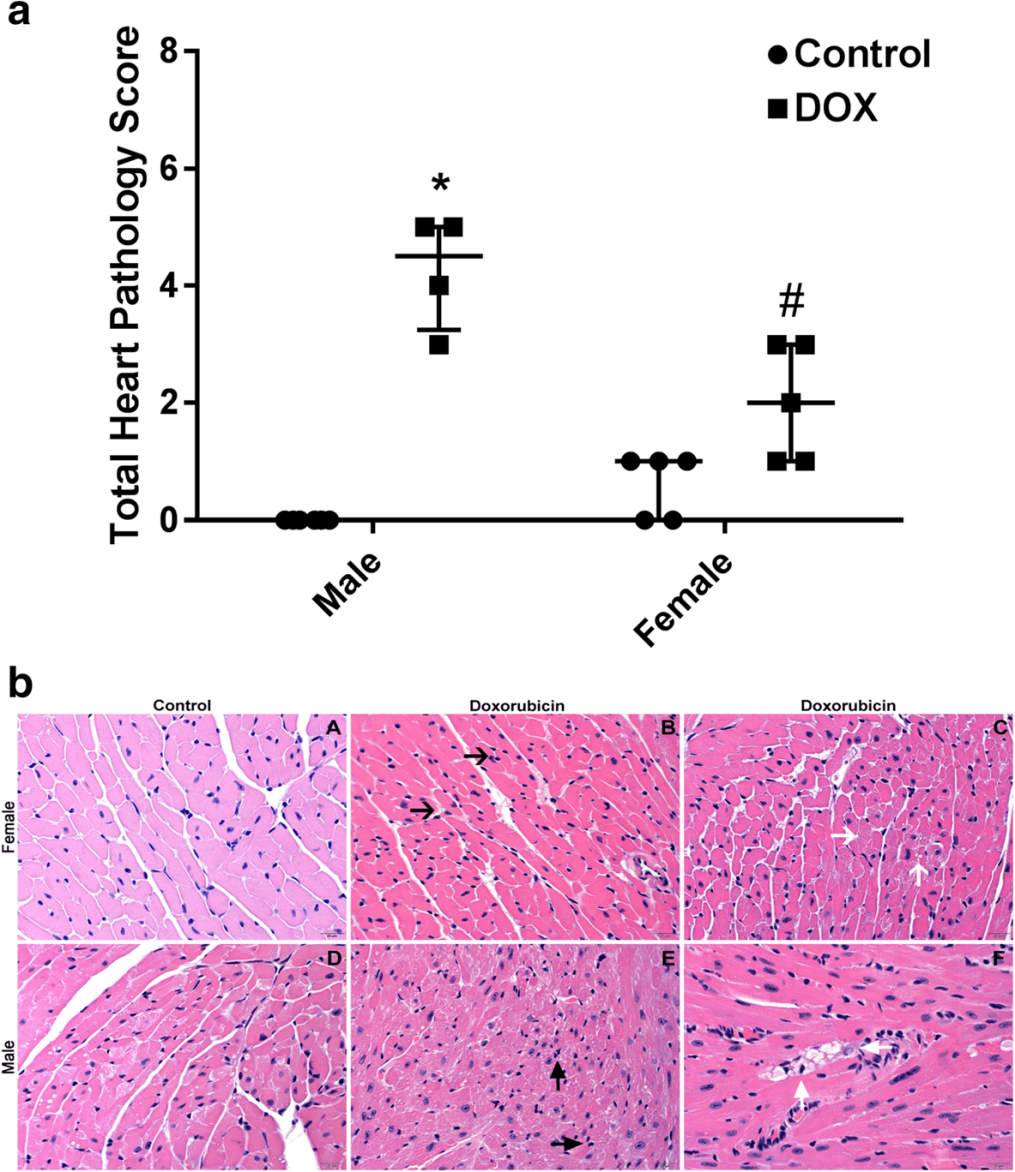
**a** Heart histopathology was assessed in heart sections stained with hematoxylin and eosin (HE) for interstitial cellularity and cardiomyocyte vacuolization and in heart sections stained with trichome for fibrosis 6 days following treatment with DOX or saline. Total heart pathology score for each animal and the median value with 95% confidence intervals shown. *p < 0.05, compared to control mice of the same sex; #*p* < 0.05, compared to male DOX-treated mice. **b** Representative photomicrographs from the HE-stained heart sections of control and DOX-treated male and female mice. In contrast to the female control mice (A, total pathology score = 1), male control mice (D, total pathology score = 0), and DOX-treated female mice (B and C, total pathology score = 3), the DOX-treated male mice (E and F, total pathology score = 5) demonstrated more severe interstitial cellularity (E, *black arrows*) and cardiomyocyte vacuolization (F, *white arrows*)

To document sex-related differences of DOX-induced cardiotoxicity in C57Bl/6 mice, acute DOX cardiotoxicity was induced by a single IP injection of 20 mg/kg DOX in male and female mice. In contrast to the female mice, all of which survived until the 6th day after DOX injection, there was a significantly increased mortality (5/9, 55%) in male mice (Additional file 3). Acute DOX-induced toxicity resulted in a significant 7 and 11% reduction in body weight 1 day after DOX injection; and 19 and 18% reduction in body weight 6 days after DOX injection, in male and female mice, respectively (Fig. 1a). Acute DOX exposure also caused a significant 6 and 7% reduction of heart weight to tibial length ratio 1 day after DOX administration, and 20 and 21% reduction of heart weight to tibial length ratio 6 days after DOX administration in male and female mice, respectively (Fig. 1b). The cardiac function of female mice and surviving male mice was assessed by trans-thoracic echocardiography on the 5th day after DOX injection. There was a trend toward lower stroke volume (Fig. 1c) in the surviving DOX-treated male mice; however, it did not reach statistical significance (*p* = 0.41). There was no significant difference in the left ventricular ejection fraction or fractional shortening between control and DOX-treated mice in either male or female mice (data not shown). Nevertheless, acute DOX cardiotoxicity caused more pronounced histopathological changes in the hearts of male than female mice (Fig. 2 and Table 1).

**Table 1:** Heart pathology scores based on evaluation of hematoxylin and eosin (HE) and trichome stained sections

Pathology		Interstitial cellularity					Vacuolization					Fibrosis					Cumulative		
Degree		0	1	2	3	4	0	1	2	3	4	0	1	2	3	4	Mean	Median	95% CI
Male	Control	6	0	0	0	0	6	0	0	0	0	6	0	0	0	0	0	0	0
	DOX	0	2	2	0	0	0	1	2	1	0	1	3	0	0	0	4.25	4.5*	3–5
Female	Control	2	3	0	0	0	5	0	0	0	0	5	0	0	0	0	0.6	1	0–1
	DOX	0	4	1	0	0	3	2	0	0	0	3	2	0	0	0	2	2^#^	1–3

*p < 0.05 compared to saline-treated mice of the same sex; ^#^ *p* < 0.05 compared to male DOX-treated mice

### Effect of acute DOX-induced cardiotoxicity on ANP and BNP

**Fig. 3:**
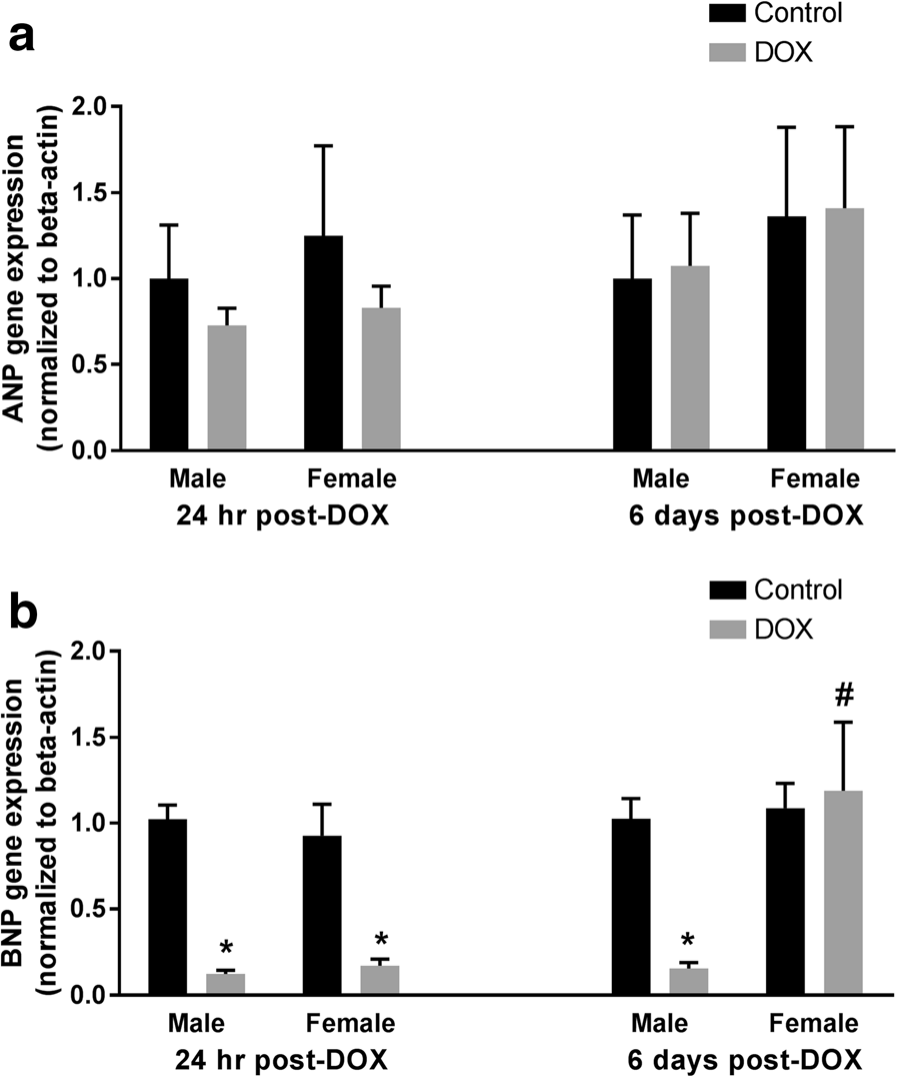
Effect of DOX exposure on ANP and BNP gene expression. Total RNA was isolated from the hearts of male or female C57Bl/6 mice collected 24 h or 6 days after the administration of a single IP injection of 20 mg/kg DOX or saline. Gene expression of **a** ANP or **b** BNP was determined by real-time PCR. Results of all groups were normalized to beta-actin levels and are expressed relative to male control of the same time point. Values are expressed as mean ± SEM. *p < 0.05, compared to control mice of the same sex; #*p* < 0.05, compared to male DOX-treated mice by Tukey’s post-hoc analysis

In order to further characterize the sexual dimorphism of acute DOX-mediated cardiotoxicity in C57Bl/6 mice, we determined the sex-dependent effect of DOX on the gene expression of the natriuretic peptides, ANP and BNP. Acute DOX-mediated cardiotoxicity did not significantly change the gene expression of ANP in either male or female animals (Fig. 3a). In contrast, DOX caused a marked inhibition of BNP expression in both sexes (Fig. 3b). At 24 h after DOX administration, gene expression of BNP was lower by 87 and 83% than sex-matched controls in the male and female mice, respectively (Fig. 3b). Of interest, 6 days after DOX administration, BNP was still inhibited by 84% in male mice, but it had returned to a control value in female mice (Fig. 3b), indicating a recovery from DOX-mediated inhibition of BNP in hearts of female mice.

### Effect of acute DOX-induced cardiotoxicity on inflammatory and apoptotic markers

**Fig. 4:**
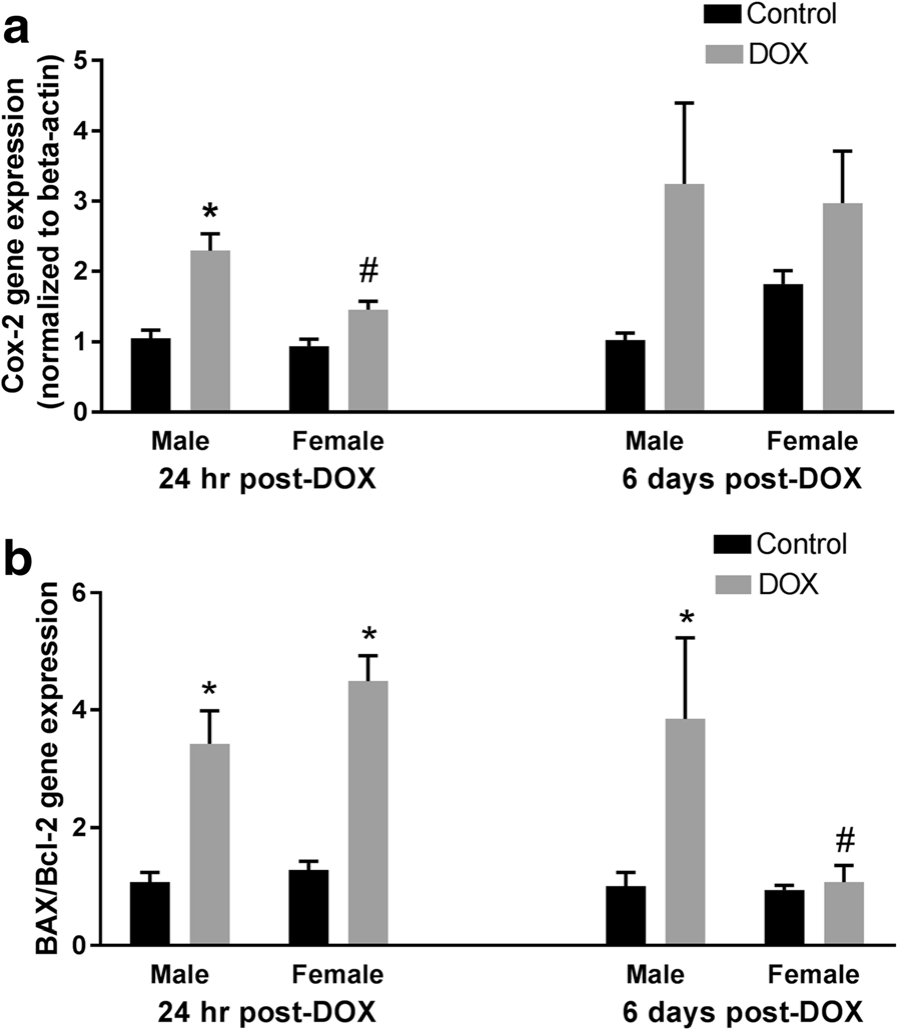
Effect of DOX exposure on COX-2 and BAX/Bcl2 gene expression. Total RNA was isolated from the hearts of male or female C57Bl/6 mice collected 24 hours or 6 days after the administration of a single IP injection of 20 mg/kg DOX or saline. Gene expression of **a** COX-2 or **b** BAX/Bcl-2 was determined by real-time PCR. Results of all groups were normalized to beta-actin levels and are expressed relative to male control of the same time point. Values are expressed as mean ± SEM. *p < 0.05, compared to control mice of the same sex; #*p* < 0.05, compared to male DOX-treated mice by Tukey’s post-hoc analysis

Since inflammation and apoptosis play important roles in the pathogenesis of acute DOX cardiotoxicity [24, 33, 34], we determined the effect of DOX on the cardiac expression of the inflammatory marker COX-2 and the apoptotic marker BAX/Bcl-2 in both male and female mice. Acute DOX exposure caused a significant 2.3-fold induction of the inflammatory marker COX-2 gene expression in the hearts of male mice 24 h after DOX administration. In a sex-dependent manner, acute DOX exposure did not cause a significant difference in COX-2 gene expression in female mice (Fig. 4a). Six days following DOX administration, there was a trend toward an increased COX-2 expression in the hearts of male mice (*p* = 0.06), but the increase did not reach statistical significance (Fig. 4a). While acute DOX exposure caused a significant 3.4- and 4.5-fold induction of BAX/Bcl-2 gene expression in the hearts of male and female mice, respectively, 24 h after DOX administration, BAX/Bcl-2 was still induced in hearts of male mice by 3.8-fold, but it returned to a control value in the hearts of female mice 6 days after DOX administration (Fig. 4b), indicating a recovery from DOX-induced apoptosis in the hearts of female mice.

### Acute DOX exposure results in sexually dimorphic alterations in CYP expression

#### Cyp1 family

**Fig. 5:**
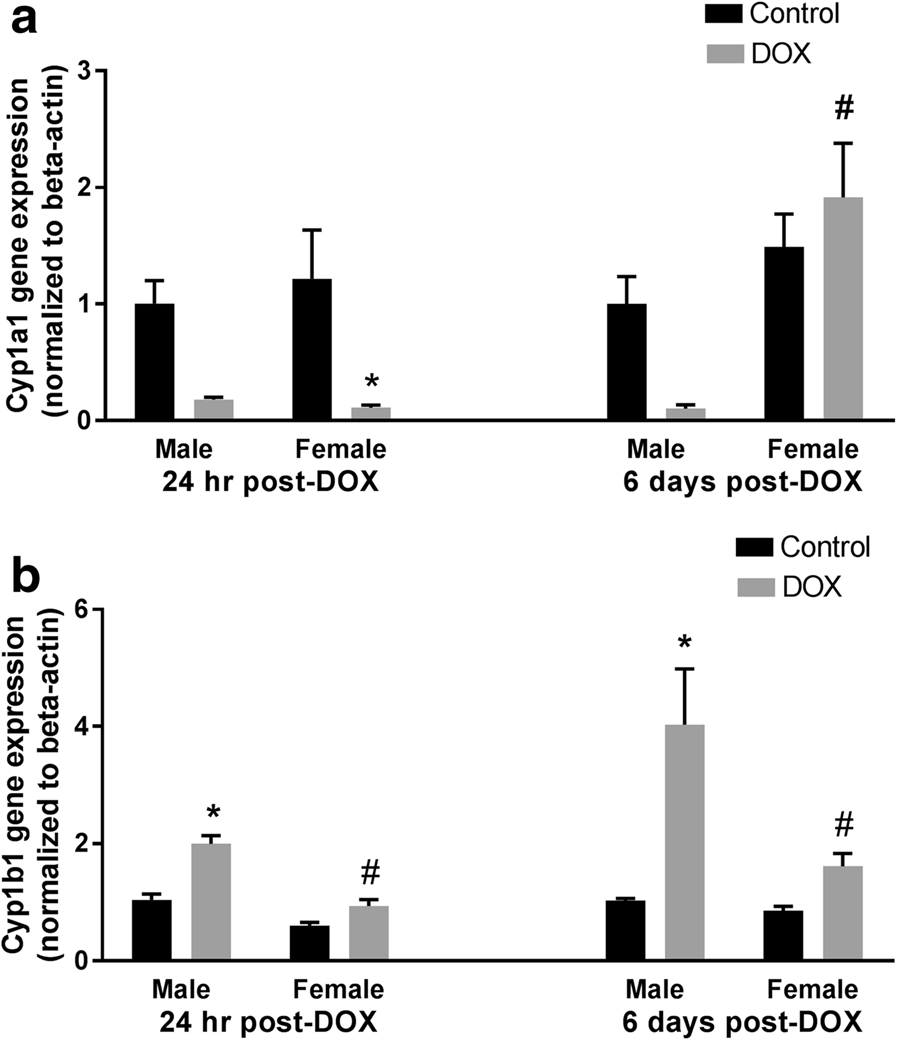
Effect of DOX exposure on gene expression of the Cyp1 family of cytochrome CYP enzymes. Total RNA was isolated from the hearts of male or female C57Bl/6 mice collected 24 h or 6 days after the administration of a single IP injection of 20 mg/kg DOX or saline. Gene expression of **a** Cyp1a1 or **b** Cyp1b1 was determined by real-time PCR. Results of all groups were normalized to beta-actin levels and are expressed relative to male control of the same time point. Values are expressed as mean ± SEM. *p < 0.05, compared to control mice of the same sex; #*p* < 0.05, compared to male DOX-treated mice by Tukey’s post-hoc analysis. Main effects are not represented statistically on the figures, and the reader can refer to Table 2 for the main effects statistics

One day following DOX injection, a greater than 80% reduction in the expression of Cyp1a1 in the heart was observed in both male and female mice (*p* value = 0.05 in males). Cyp1a1 gene expression returned to a control value in females, but not in males, 6 days post-DOX (Fig. 5a), demonstrating a significant sex effect (Table 2 and Additional file 5). In contrast to Cyp1a1, acute DOX exposure caused a significant twofold induction of Cyp1b1 gene expression in the hearts of male mice 1 day after DOX injection. A further increase (fourfold) was observed 6 days after DOX injection. Of interest, DOX toxicity did not alter Cyp1b1 gene expression in the female heart (Fig. 5b) as there was a significant interaction between sex and DOX exposure 1 day post-DOX and a significant sex effect 6 days post-DOX (Table 2 and Additional files 4 and 5).

**Table 2:** *q* values for the effect of DOX, sex, and the interaction between sex and DOX in regulating CYP genes expression based on a 2-way ANOVA analysis and a 5% false discovery rate

	1 day post-DOX			6 days post-DOX		
	DOX effect	Sex effect	Interaction effect	DOX effect	Sex effect	Interaction effect
Cyp1a1	0.0001*	0.3346	0.3865	0.4935	0.0064*	0.1977
Cyp1b1	<0.0001*	<0.0001*	0.0200*	0.0013*	0.0064*	0.0831
Cyp2c29	0.0012*	0.0006*	0.0200*	0.86	0.0433*	1.0000
Cyp2c44	0.0013*	0.051	0.1048	0.08	0.0064*	0.9700
Cyp2e1	<0.0001*	0.0164*	0.0364*	0.08	0.5371	1.0000
Cyp2j9	<0.0001*	0.2994	0.0567	0.2812	0.0727	1.0000
Cyp4a10	<0.0001*	0.0009*	0.3627	0.0943	0.1776	1.0000

Specific post-hoc testing results can be found on the relevant figures

*q < 0.05 denotes discoveries

#### Cyp2 family

**Fig. 6:**
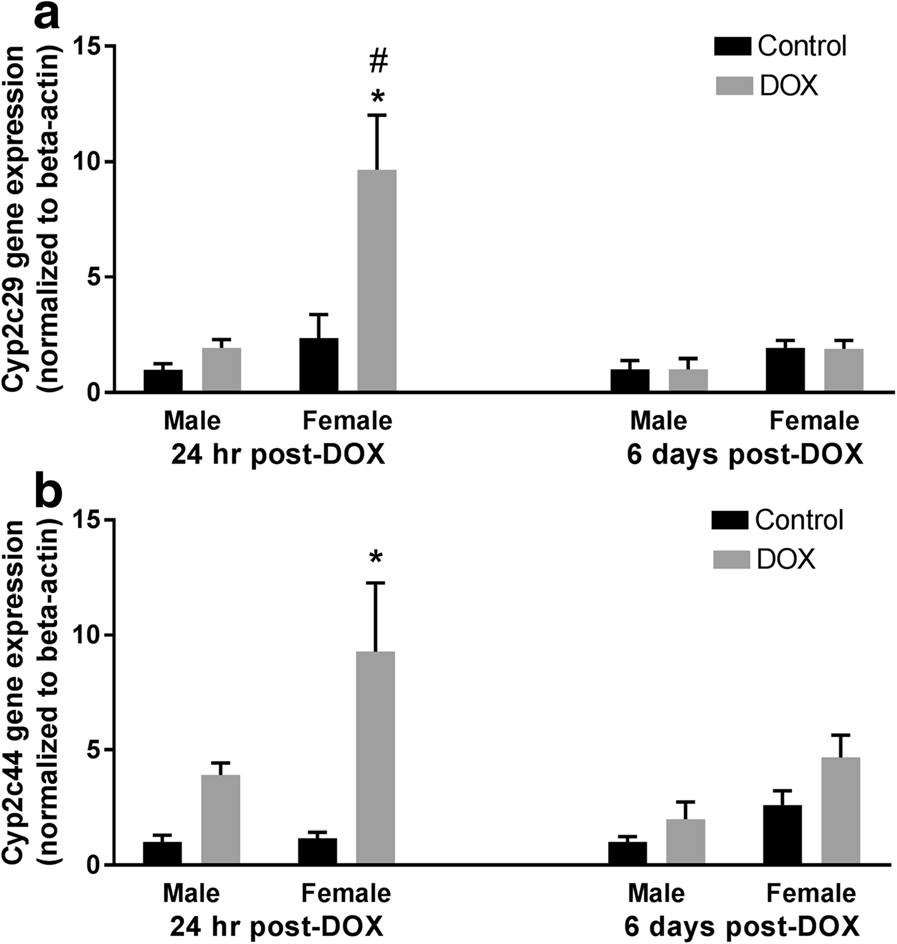
Effect of DOX exposure on gene expression of the Cyp2c family of cytochrome CYP enzymes. Total RNA was isolated from the hearts of male or female C57Bl/6 mice collected 24 h or 6 days after the administration of 20 mg/kg DOX or saline. Gene expression of **a** Cyp2c29 or **b** Cyp2c44 was determined by real-time PCR. Results of all groups were normalized to beta-actin levels and are expressed relative to male control of the same time point. Values are expressed as mean ± SEM. *p < 0.05, compared to control mice of the same sex; #*p* < 0.05, compared to male DOX-treated mice by Tukey’s post-hoc analysis. Main effects are not represented statistically on the figures, and the reader can refer to Table 2 for the main effects statistics

**Fig. 7:**
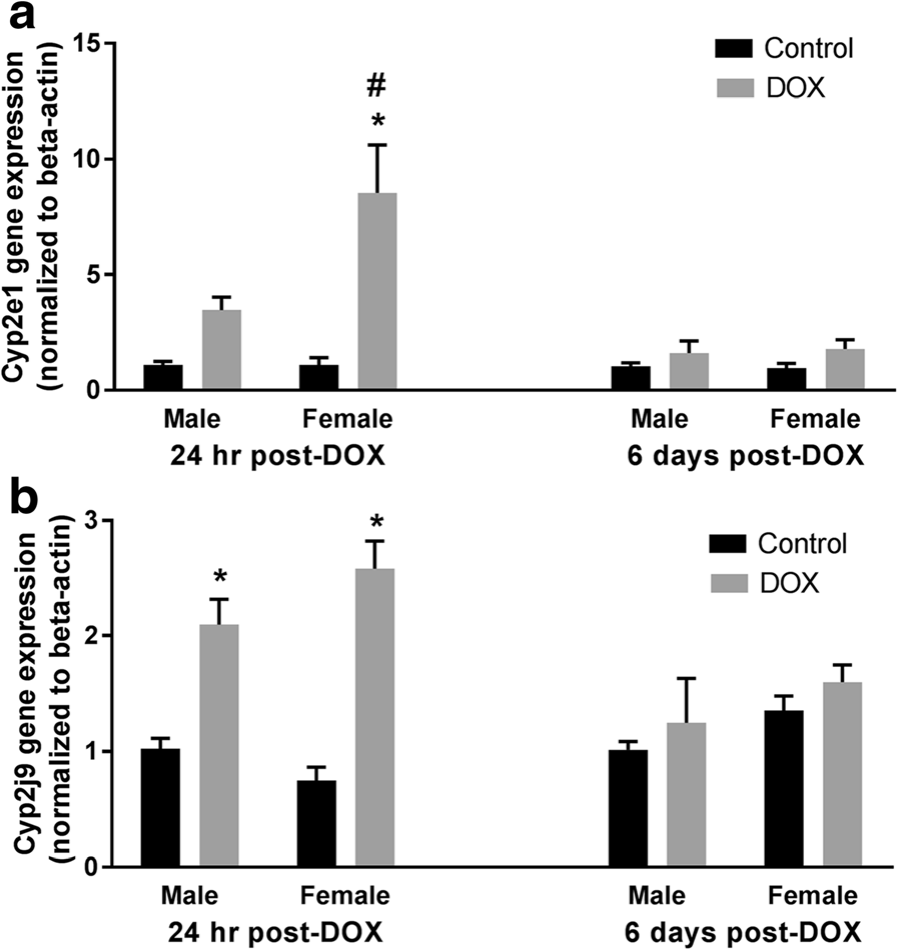
Effect of DOX exposure on Cyp2e1 and Cyp2j9 gene expression. Total RNA was isolated from the hearts of male or female C57Bl/6 mice collected 24 h or 6 days after the administration of 20 mg/kg DOX or saline. Gene expression of **a** Cyp2e1 or **b** Cyp2j9 was determined by real-time PCR. Results of all groups were normalized to beta-actin levels and are expressed relative to male control of the same time point. Values are expressed as mean ± SEM. *p < 0.05, compared to control mice of the same sex; #*p* < 0.05, compared to male DOX-treated mice by Tukey’s post-hoc analysis. Main effects are not represented statistically on the figures, and the reader can refer to Table 2 for the main effects statistics

Expression of Cyp2c29 and Cyp2c44 was increased by twofold and eightfold, respectively, by DOX in the hearts of female animals 1 day after treatment. While there was no significant change in Cyp2c29 gene expression in the hearts of male mice at 24 h or 6 days after DOX administration, acute DOX exposure caused a non-significant fourfold induction of Cyp2c44 in hearts of male mice 24 h after DOX administration (*p* = 0.54) (Fig. 6a, b). Additionally, there was a significant interaction between sex and DOX exposure in Cyp2c29 gene expression 24 h post-DOX (Table 2 and Additional file 4). A greater than eightfold induction of Cyp2e1 gene expression was observed in the hearts of female mice 1 day post-DOX, which returned to a control value 6 days after treatment (Fig. 7a). A non-significant 3.4-fold induction in Cyp2e1 gene expression was observed in the heart of male mice (*p* = 0.35), demonstrating a significant interaction between sex and DOX exposure 24 h post-DOX (Table 2 and Additional file 4). Acute DOX exposure caused a significant twofold and threefold induction of Cyp2j9 gene expression 1 day following DOX treatment in the heart of male and female mice, respectively. In both sexes, there was no significant difference in Cyp2j9 gene expression between control and DOX-treated animals 6 days after DOX administration (Fig. 7b).

#### Cyp4 family

**Fig. 8:**
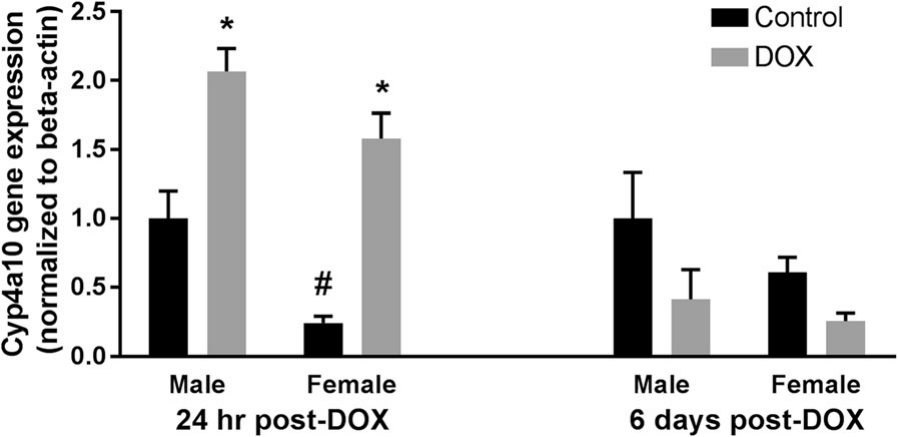
Effect of DOX exposure on Cyp4a10 gene expression. Total RNA was isolated from the hearts of male or female C57Bl/6 mice collected 24 h or 6 days after the administration of 20 mg/kg DOX or saline. Gene expression of Cyp4a10 was determined by real-time PCR. Results of all groups were normalized to beta-actin levels and are expressed relative to male control of the same time point. Values are expressed as mean ± SEM. *p < 0.05, compared to control mice of the same sex; #*p* < 0.05, compared to control male mice by Tukey’s post-hoc analysis. Main effects are not represented statistically on the figures, and the reader can refer to Table 2 for the main effects statistics

Acute exposure to DOX significantly increased Cyp4a10 mRNA levels in hearts of both male and female mice (twofold and sixfold induction, respectively) 1 day after DOX administration. This was reversed in both sexes 6 days after treatment (Fig. 8). Interestingly, Cyp4a10 gene expression was lower in the hearts of female control mice than in male control mice in the 24 hour cohort (Fig. 8) with a significant effect of sex on Cyp4a10 expression (Table 2 and Additional file 4). While the Cyp4a10 gene expression was lower in the hearts of female control mice than in male control mice in the 6 days cohort as well, the difference was not statistically significant (*p* = 0.6).

## Discussion

Sex-related differences in DOX-induced cardiotoxicity have been well documented in several rat species, including Wistar rats [12, 14], spontaneously hypertensive rats (SHR) [11], and spontaneously hypertensive heart failure-prone rats (SHHF) [15]. Recently, sexual dimorphism of chronic DOX-induced cardiotoxicity has also been reported in B6C3F1 mice [16]. However, sexual dimorphism of acute DOX-induced cardiotoxicity has never been shown in mice, despite the ubiquitous use of mice as a model for acute DOX-induced cardiotoxicity [35–37]. Therefore, in the current work, we demonstrate for the first time a significant sexual dimorphism of acute DOX-induced cardiotoxicity in one of the most commonly used mouse strains, the C57Bl/6 mouse. Acute cardiotoxicity in adult mice was induced by a single IP injection of 20 mg/kg DOX. This dose has been previously used to induce acute cardiotoxicity in adult male mice in several studies [35, 37]. Similar to these previous studies, DOX administration caused significant toxicity in male mice in our study as demonstrated by significant mortality, a decrease in heart and body weights, and cardiac histopathological lesions. Similar to previous studies, female mice are protected against several features of DOX-induced toxicity [11, 12, 14–16]. Indeed, there was no mortality and milder histopathological lesions in female mice. However, a decrease in body and heart weights was observed in both male and female mice. These results corroborate the findings of a previous study where chronic DOX treatment caused a significant weight loss in both male and female Wistar rats, with 50% mortality only in male rats [12]. It is important to mention that the observed mortality in male mice can also be attributed to acute DOX-induced multi-organ toxicity including bone marrow toxicity, gastrointestinal toxicity, nephrotoxicity, and/or hepatotoxicity [38–40]. Determining sex-related differences of acute DOX-induced toxicity in these organs warrants further research.

To further document the observed sexual dimorphism, we measured the gene expression of the natriuretic peptides, ANP and BNP, the inflammatory marker, COX-2, and the apoptotic marker, BAX/Bcl-2, in the hearts of male and female mice. In the current study, acute DOX-induced cardiotoxicity caused a marked inhibition in the expression of BNP, but not ANP, 24 h following DOX administration in both male and female mice. In agreement with our findings, DOX selectively inhibited BNP versus ANP in cultured neonatal rat cardiomyocytes [41]. Similarly, we have previously shown that acute DOX cardiotoxicity caused an earlier and a more pronounced inhibition of BNP as compared to ANP in the hearts of male Sprague Dawley rats [19]. The inhibition of BNP by acute DOX cardiotoxicity can be attributed to the DOX suppression of GATA-4 [42] which is known to regulate BNP expression [43]. Of importance, a marked sex-dependent difference in BNP gene expression was found 6 days after DOX administration. The gene expression of BNP is markedly inhibited in the hearts of DOX-treated male mice, but not in DOX-treated female mice. Since apoptosis is known to play an important role in mediating acute DOX-induced cardiotoxicity [24], we measured the gene expression of the apoptotic marker, BAX/Bcl-2. Acute DOX cardiotoxicity has been previously demonstrated to induce BAX/Bcl-2 gene expression in the hearts of male mice [44]. In our current work, we also demonstrate that acute DOX exposure caused a significant induction of BAX/Bcl-2 gene expression in the hearts of both male and female mice 24 h after DOX administration. However, in a sex-dependent manner, BAX/Bcl-2 gene expression returned to control values 6 days after DOX injection in the hearts of female, but not male mice. The early marked inhibition of BNP and induction of BAX/Bcl-2 in both male and female mice may imply that both sexes had been exposed to equal DOX-induced early injury, but the females have certain compensatory mechanisms that allowed them to recover from this initial injury and relieve both DOX-mediated inhibition of BNP and DOX-induced apoptosis 6 days later. The recovery from DOX-mediated inhibition of BNP in the hearts of female mice may indicate a relief from DOX-induced suppression of GATA-4. In contrast to BNP and BAX/Bcl2, DOX-mediated cardiotoxicity increased the expression of COX-2 in the hearts of male, but not female mice 24 h after DOX administration, indicating a potential role of inflammation in mediating the early pathology of acute DOX-induced cardiotoxicity in male mice.

A few mechanisms have been previously proposed to explain the sexual dimorphism of DOX-induced cardiotoxicity, including sex-related differences in cardiac energy metabolism [12], cardiolipin remodeling [14], and mast cell activity [11]. Several investigators attributed this sexual dimorphism to the cardioprotection conferred by estrogen in female experimental animals [11, 13, 45]. Intriguingly, another study showed that the expression of carbonyl reductase, a major DOX-metabolizing enzyme, is higher in the kidney and liver of female than in male mice [46]. The authors suggested that higher expression of carbonyl reductase may make female mice more sensitive to DOX-induced toxicity; however, DOX-induced cardiotoxicity was not actually assessed in their study [46]. Therefore, the underlying mechanisms of sexual dimorphism in DOX-induced toxicity are still poorly understood. A growing body of evidence suggests an important role of CYP enzymes in the pathogenesis of DOX-induced cardiotoxicity [19, 47, 48]. CYP enzymes play important roles in metabolizing sex steroids and other endogenous compounds, such as arachidonic acid that have significant biological effects on the cardiovascular system [20]. Interestingly, there is a significant sex-divergent expression of several CYP enzymes in both humans and experimental animals [49]. Therefore, we hypothesized that DOX alters CYP enzymes in a sex-dependent manner to mediate sexual dimorphism of DOX-induced cardiotoxicity.

In the current study, acute DOX exposure caused a marked inhibition of Cyp1a1 gene expression in the hearts of male and female mice 1 day after DOX injection. In contrast to this finding, we have previously reported that acute DOX-induced cardiotoxicity caused significant induction of CYP1A1 in the hearts of male Sprague Dawley rats [19]. Similarly, a single 10 mg/kg DOX injection caused significant induction of Cyp1a1 in the hearts of 8-week-old male C57Bl/6 mice [50]. In order to confirm our current finding, we used three different sets of Cyp1a1 primers selected from three different studies (Additional file 2). The three different experiments resulted in the same finding, confirming the inhibition of Cyp1a1 gene expression in this study. Therefore, the discrepancy may be due to the difference in the dose of DOX and/or the age of mice. DOX-mediated inhibition of Cyp1a1 may be attributed to DOX-induced inflammation, since inflammation has been shown to downregulate Cyp1a1 in the hearts of male C57Bl/6 mice [51]. However, this notion cannot explain the inhibition of Cyp1a1 in the hearts of female mice, since they had minimal inflammation. Of interest, the gene expression of Cyp1a1 returned to a control value 6 days after DOX administration in female, but not in male mice, indicating the recovery of the female hearts from the early DOX-induced injury.

DOX administration caused a significant induction of Cyp1b1 in the hearts of male mice 24 h and 6 days after DOX injection, but not in the hearts of female mice. In agreement with this finding, we have previously reported DOX-mediated induction of CYP1B1 in the heart of male SD rats [19], and in the cardiac-derived H9c2 cells [18]. The male-specific induction of Cyp1b1 by DOX may play an important role in mediating the sexual dimorphism of DOX-induced cardiotoxicity. In male rodents, Cyp1b1 has been shown to mediate DOX-induced cardiotoxicity via the metabolism of arachidonic acid to the cardiotoxic mid-chain hydroxyeicosatetraenoic acid metabolites [48], and hypertension-associated cardiomyopathy via the metabolism of testosterone to the more cardiotoxic metabolite, 6β-hydroxytestosterone [52]. Inhibition of CYP1B1 in the two previous studies conferred significant protection against both DOX-induced cardiotoxicity and hypertension-associated cardiomyopathy [48, 52].

We have previously shown that acute DOX-induced cardiotoxicity causes significant induction of CYP2C11 in the hearts of male Sprague Dawley rats [19]. In the present study, DOX administration caused a modest non-statistically significant induction of Cyp2c44 24 h after DOX injection in the hearts of male mice. On the other hand, acute DOX exposure caused a marked induction of Cyp2c29 and Cyp2c44 gene expression in the heart of female mice 24 h after DOX administration. Cyp2c enzymes are important epoxygenases which may play a role in protecting the female heart by producing cardioprotective metabolites such as the epoxyeicosatrienoic acids [53]. Sun et al. have found that shear stress upregulated Cyp2c29 expression and increased the protective epoxyeicosatrienoic acids in female mice by an estrogen-dependent mechanism [54]. Interestingly, ovariectomized female rats have been shown to be as sensitive as male rats to DOX-induced cardiotoxicity [11], which further confirms the protective role of estrogen against DOX-induced cardiotoxicity.

Similar to Cyp2c29, acute DOX-induced cardiotoxicity induced the gene expression of Cyp2e1 only in the hearts of female mice 24 h following DOX administration. In agreement with our current findings in male mice, we have previously demonstrated that acute DOX cardiotoxicity did not change CYP2E1 expression in the hearts of male Sprague Dawley rats [19]. However, DOX induced CYP2E1 in a concentration-dependent manner in H9c2 cardiomyoblasts in vitro [18]. Despite the well-documented role of hepatic Cyp2e1 in mediating alcohol-induced hepatotoxicity [55], the role of cardiac Cyp2e1 is not well studied. Pharmacological inhibition and genetic knock down of Cyp2e1 have been shown to confer cardioprotection in a DOX-induced dilated cardiomyopathy mouse model by inhibiting oxidative stress and apoptosis [56]. Nevertheless, Cyp2e1 metabolizes arachidonic acid to 19-hydroxyeicosatetraenoic acid which has cardioprotective properties [57]. Therefore, further research is needed to confirm whether cardiac Cyp2e1 is protective or detrimental to the heart. Similar to our previous studies in male Sprague Dawley rats [19] and H9c2 cardiomyoblasts [18], acute DOX cardiotoxicity increased Cyp2j9 gene expression in the hearts of both male and female mice. Cyp2j9 is an omega-1 hydroxylase enzyme that metabolizes arachidonic acid to the presumably cardioprotective 19-hydroxyeicosatetraenoic acid [58]. Therefore, induction of Cyp2j9 by DOX can be an adaptive mechanism to protect the heart from further DOX-induced toxicity.

Several studies have investigated the sexual divergence of Cyp4a10 expression in the kidney and liver of different strains of mice. In one study, there was no sex-dependent difference in the expression of Cyp4a10 in the kidney of C57Bl/6 mice [59]. In another study, Cyp4a10 was expressed at a higher level in the liver and kidney of female than in male C57Bl/6 mice [21]. To the best of our knowledge, there is no information about sex-related differences in Cyp4a10 expression in the mouse heart. In the current study, we found that the expression of Cyp4a10 was significantly lower in the hearts of control female than in control male mice in the 24-h cohort, suggesting organ-specific sexual divergence in Cyp4a10 expression. Although the expression of Cyp4a10 in the hearts of female mice was 60% of its expression in male mice in the 6-day cohort, this difference was not statistically significant. There are three possibilities that may explain this controversy: first, control mice are not “naïve” control mice i.e., they were administered an IP injection of sterile saline in equal volumes to DOX injections. These injections with the associated stress may modulate the sex effect at the two different time points (24 h and 6 days after the saline injection). Second, the 6-day mice had undergone echocardiography under isoflurane anesthesia which may have also modulated this sex effect. Finally, this difference may just be due to different statistical power, since the number of animals in the 24-h cohort was higher than that of the 6-day cohort allowing for more power to detect differences. Lower expression of cardiac Cyp4a10 may contribute to the overall protection of females against heart disease as Cyp4a10 has been shown to metabolize arachidonic acid to the cardiotoxic metabolite 20-hydroxyeicosatetraenoic acid [59]. Despite a lower constitutive expression of Cyp4a10 in the female hearts, acute DOX-induced cardiotoxicity caused a marked induction of Cyp4a10 in the hearts of both male and female mice, suggesting that this enzyme may not play an important role in the sexual dimorphism of DOX-induced cardiotoxicity. In agreement with previous studies [60, 61], the mRNA levels of Cyp4a12 and Cyp4a14 were below the levels of detection in the hearts of both male and female mice in the current study (data not shown).

## Conclusions

**Fig. 9:**
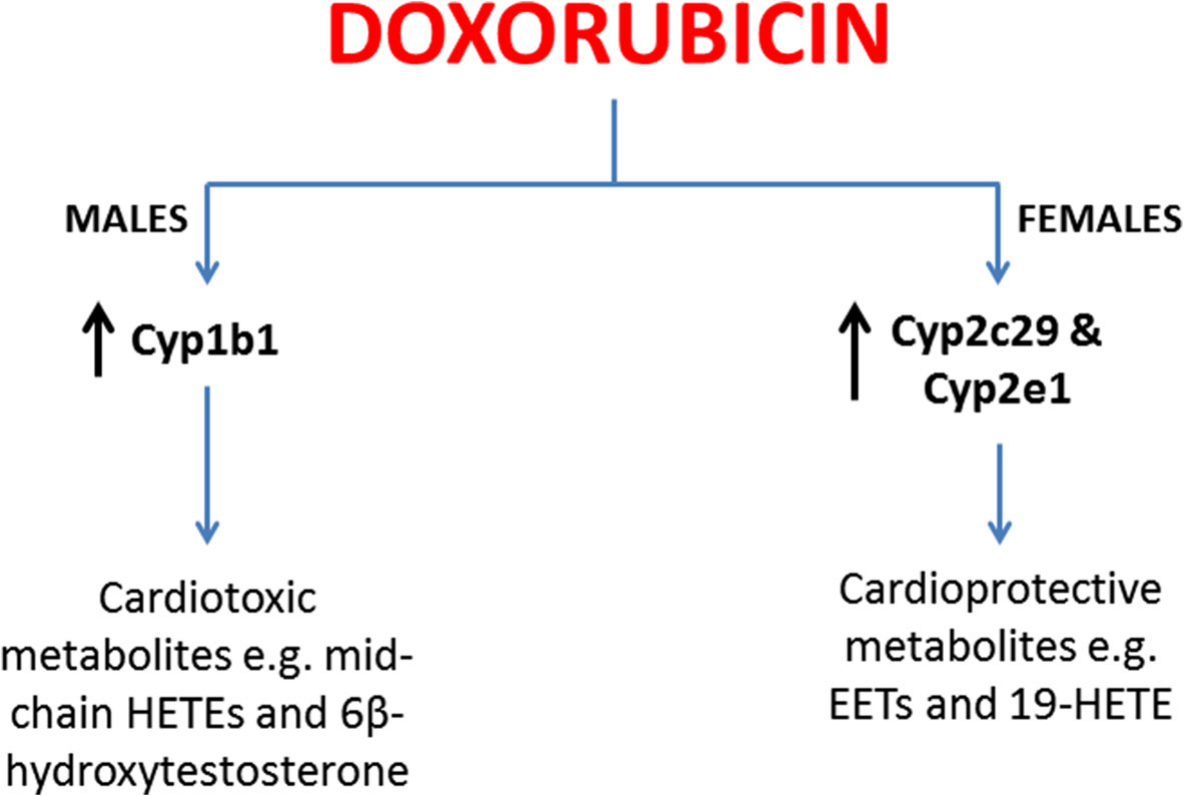
Sex-dependent alteration of cardiac cytochrome CYP gene expression by doxorubicin. Doxorubicin causes male-specific induction of Cyp1b1 gene expression. Cyp1b1 enzyme is known to generate cardiotoxic metabolites such as mid-chain hydroxyeicosatetraenoic acids (HETEs) and/or 6β-hydroxytestosterone. On the other hand, DOX causes female-specific induction of Cyp2c29 and Cyp2e1 gene expression. Cyp2c29 and Cyp2e1 enzymes are known to generate cardioprotective metabolites such as epoxyeicosatrienoic acids (EETs) and 19-hydroxyeicosatetraenoic acid (19-HETE)

In conclusion, we have identified significant sex-related differences in acute DOX-induced cardiotoxicity in adult C57Bl/6 mice. These findings corroborate previous studies that demonstrated marked sexual dimorphism of acute and chronic DOX-induced cardiotoxicity in several rat strains. Since the C57Bl/6 mouse is a popular model in studies of acute DOX-induced cardiotoxicity, characterizing these sex-related differences is very important to guide researchers to properly interpret the results obtained from female mouse models of acute DOX-induced cardiotoxicity. In addition, we have demonstrated for the first time that acute DOX exposure alters CYP gene expression in a sex-dependent manner. As illustrated in Fig. 9, significant male-specific induction of Cyp1b1 and female-specific induction of Cyp2c29 and Cyp2e1 may play important roles in mediating sex-related differences of DOX-induced cardiotoxicity. A limitation to this study, however, is that we only measured the gene expression of these enzymes. Therefore, the protein expression and activity toward metabolism of different substrates are to be confirmed in future studies.

## Additional files

Additional file 1: Representative RIN values. The quality of the extracted RNA was determined by measuring the RIN values using an Agilent 2100 Bioanalyzer (Santa Clara, CA). (DOCX 111 kb)
Additional file 2: Primer sequences used in the current study. (DOCX 15 kb)
Additional file 3: Kaplan-Meier survival curve for male (solid line, *n* = 9) or female (dashed line, *n* = 5) mice following a single intraperitoneal injection of 20 mg/kg doxorubicin (DOX). **p* < 0.05 (DOCX 57 kb)
Additional file 4: The effect of DOX, sex, and the interaction between sex and DOX in regulating Cytochrome P450 gene expression after 24 h of administering a single intraperitoneal injection of DOX 20 mg/kg. (DOCX 15 kb)
Additional file 5: The effect of DOX, sex, and the interaction between sex and DOX in regulating Cytochrome P450 gene expression after 6 days of administering a single intraperitoneal injection of DOX 20 mg/kg. (DOCX 15 kb)

## Abbreviations

ANP: Atrial natriuretic peptide
BAX: Bcl-2 associated X protein
Bcl-2: B-cell lymphoma 2
BNP: B-type natriuretic peptide
Cox-2: Cyclooxygenase-2
CYP: Cytochrome P450
DOX: Doxorubicin

## References

[1] Myrehaug S, Pintilie M, Tsang R, Mackenzie R, Crump M, Chen Z, Sun A, Hodgson DC (2008). Cardiac morbidity following modern treatment for Hodgkin lymphoma: supra-additive cardiotoxicity of doxorubicin and radiation therapy. Leuk Lymphoma.

[2] Conway A, McCarthy AL, Lawrence P, Clark RA (2015). The prevention, detection and management of cancer treatment-induced cardiotoxicity: a meta-review. BMC Cancer.

[3] Takemura G, Fujiwara H (2007). Doxorubicin-induced cardiomyopathy from the cardiotoxic mechanisms to management. Prog Cardiovasc Dis.

[4] Lipshultz SE, Sambatakos P, Maguire M, Karnik R, Ross SW, Franco VI, Miller TL (2014). Cardiotoxicity and cardioprotection in childhood cancer. Acta Haematol.

[5] Lotrionte M, Biondi-Zoccai G, Abbate A, Lanzetta G, D’Ascenzo F, Malavasi V, Peruzzi M, Frati G, Palazzoni G (2013). Review and meta-analysis of incidence and clinical predictors of anthracycline cardiotoxicity. Am J Cardiol.

[6] Silber JH, Jakacki RI, Larsen RL, Goldwein JW, Barber G (1993). Increased risk of cardiac dysfunction after anthracyclines in girls. Med Pediatr Oncol.

[7] Lipshultz SE, Lipsitz SR, Mone SM, Goorin AM, Sallan SE, Sanders SP, Orav EJ, Gelber RD, Colan SD (1995). Female sex and drug dose as risk factors for late cardiotoxic effects of doxorubicin therapy for childhood cancer. N Engl J Med.

[8] Hequet O, Le QH, Moullet I, Pauli E, Salles G, Espinouse D, Dumontet C, Thieblemont C, Arnaud P, Antal D (2004). Subclinical late cardiomyopathy after doxorubicin therapy for lymphoma in adults. J Clin Oncol.

[9] Myrehaug S, Pintilie M, Yun L, Crump M, Tsang RW, Meyer RM, Sussman J, Yu E, Hodgson DC (2010). A population-based study of cardiac morbidity among Hodgkin lymphoma patients with preexisting heart disease. Blood.

[10] Hershman DL, McBride RB, Eisenberger A, Tsai WY, Grann VR, Jacobson JS (2008). Doxorubicin, cardiac risk factors, and cardiac toxicity in elderly patients with diffuse B-cell non-Hodgkin’s lymphoma. J Clin Oncol.

[11] Zhang J, Knapton A, Lipshultz SE, Cochran TR, Hiraragi H, Herman EH (2014). Sex-related differences in mast cell activity and doxorubicin toxicity: a study in spontaneously hypertensive rats. Toxicol Pathol.

[12] Moulin M, Piquereau J, Mateo P, Fortin D, Rucker-Martin C, Gressette M, Lefebvre F, Gresikova M, Solgadi A, Veksler V (2015). Sexual dimorphism of doxorubicin-mediated cardiotoxicity: potential role of energy metabolism remodeling. Circ Heart Fail.

[13] Gonzalez Y, Pokrzywinski KL, Rosen ET, Mog S, Aryal B, Chehab LM, Vijay V, Moland CL, Desai VG, Dickey JS (2015). Reproductive hormone levels and differential mitochondria-related oxidative gene expression as potential mechanisms for gender differences in cardiosensitivity to Doxorubicin in tumor-bearing spontaneously hypertensive rats. Cancer Chemother Pharmacol.

[14] Moulin M, Solgadi A, Veksler V, Garnier A, Ventura-Clapier R, Chaminade P (2015). Sex-specific cardiac cardiolipin remodelling after doxorubicin treatment. Biol Sex Differ.

[15] Zordoky BN, Radin MJ, Heller L, Tobias A, Matise I, Apple FS, McCune SA, Sharkey LC (2016). The interplay between genetic background and sexual dimorphism of doxorubicin-induced cardiotoxicity. Cardio-Oncol.

[16] Jenkins GR, Lee T, Moland CL, Vijay V, Herman EH, Lewis SM, Davis KJ, Muskhelishvili L, Kerr S, Fuscoe JC (2016). Sex-related differential susceptibility to doxorubicin-induced cardiotoxicity in B6C3F1 mice. Toxicol Appl Pharmacol.

[17] Segredo MP, Salvadori DM, Rocha NS, Moretto FC, Correa CR, Camargo EA, de Almeida DC, Reis RA, Freire CM, Braz MG (2014). Oxidative stress on cardiotoxicity after treatment with single and multiple doses of doxorubicin. Hum Exp Toxicol.

[18] Zordoky BN, El-Kadi AO (2008). Induction of several cytochrome P450 genes by doxorubicin in H9c2 cells. Vascul Pharmacol.

[19] Zordoky BN, Anwar-Mohamed A, Aboutabl ME, El-Kadi AO (2010). Acute doxorubicin cardiotoxicity alters cardiac cytochrome P450 expression and arachidonic acid metabolism in rats. Toxicol Appl Pharmacol.

[20] Zordoky BN, El-Kadi AO (2008). Modulation of cardiac and hepatic cytochrome P450 enzymes during heart failure. Curr Drug Metab.

[21] Renaud HJ, Cui JY, Khan M, Klaassen CD (2011). Tissue distribution and gender-divergent expression of 78 cytochrome P450 mRNAs in mice. Toxicol Sci.

[22] Zhang F, Yu X, He C, Ouyang X, Wu J, Li J, Zhang J, Duan X, Wan Y, Yue J (2015). Effects of sexually dimorphic growth hormone secretory patterns on arachidonic acid metabolizing enzymes in rodent heart. Toxicol Appl Pharmacol.

[23] Mackay B, Ewer MS, Carrasco CH, Benjamin RS (1994). Assessment of anthracycline cardiomyopathy by endomyocardial biopsy. Ultrastruct Pathol.

[24] Mantawy EM, El-Bakly WM, Esmat A, Badr AM, El-Demerdash E (2014). Chrysin alleviates acute doxorubicin cardiotoxicity in rats via suppression of oxidative stress, inflammation and apoptosis. Eur J Pharmacol.

[25] Amara IE, Elshenawy OH, Abdelrady M, El-Kadi AO (2014). Acute mercury toxicity modulates cytochrome P450, soluble epoxide hydrolase and their associated arachidonic acid metabolites in C57Bl/6 mouse heart. Toxicol Lett.

[26] Nteeba J, Ortinau LC, Perfield JW, Keating AF (2013). Diet-induced obesity alters immune cell infiltration and expression of inflammatory cytokine genes in mouse ovarian and peri-ovarian adipose depot tissues. Mol Reprod Dev.

[27] Patel A, Zhang S, Paramahamsa M, Jiang W, Wang L, Moorthy B, Shivanna B (2015). Leflunomide induces pulmonary and hepatic CYP1A enzymes via aryl hydrocarbon receptor. Drug Metab Dispos.

[28] Elshenawy OH, Anwar-Mohamed A, Abdelhamid G, El-Kadi AO (2013). Murine atrial HL-1 cell line is a reliable model to study drug metabolizing enzymes in the heart. Vascul Pharmacol.

[29] Xu W, Guo G, Li J, Ding Z, Sheng J, Li J, Tan W (2016). Activation of Bcl-2-caspase-9 apoptosis pathway in the testis of asthmatic mice. PLoS One.

[30] Alvarez-Erviti L, Seow Y, Yin H, Betts C, Lakhal S, Wood MJ (2011). Delivery of siRNA to the mouse brain by systemic injection of targeted exosomes. Nat Biotechnol.

[31] Ye J, Coulouris G, Zaretskaya I, Cutcutache I, Rozen S, Madden TL (2012). Primer-BLAST: a tool to design target-specific primers for polymerase chain reaction. BMC Bioinformatics.

[32] Livak KJ, Schmittgen TD (2001). Analysis of relative gene expression data using real-time quantitative PCR and the 2(-delta delta C (T)) method. Methods.

[33] Pecoraro M, Del Pizzo M, Marzocco S, Sorrentino R, Ciccarelli M, Iaccarino G, Pinto A, Popolo A (2016). Inflammatory mediators in a short-time mouse model of doxorubicin-induced cardiotoxicity. Toxicol Appl Pharmacol.

[34] Arola OJ, Saraste A, Pulkki K, Kallajoki M, Parvinen M, Voipio-Pulkki LM (2000). Acute doxorubicin cardiotoxicity involves cardiomyocyte apoptosis. Cancer Res.

[35] Zhu W, Soonpaa MH, Chen H, Shen W, Payne RM, Liechty EA, Caldwell RL, Shou W, Field LJ (2009). Acute doxorubicin cardiotoxicity is associated with p53-induced inhibition of the mammalian target of rapamycin pathway. Circulation.

[36] Soni H, Pandya G, Patel P, Acharya A, Jain M, Mehta AA (2011). Beneficial effects of carbon monoxide-releasing molecule-2 (CORM-2) on acute doxorubicin cardiotoxicity in mice: role of oxidative stress and apoptosis. Toxicol Appl Pharmacol.

[37] Li K, Sung RY, Huang WZ, Yang M, Pong NH, Lee SM, Chan WY, Zhao H, To MY, Fok TF (2006). Thrombopoietin protects against in vitro and in vivo cardiotoxicity induced by doxorubicin. Circulation.

[38] Cardani D, Sardi C, La Ferla B, D’Orazio G, Sommariva M, Marcucci F, Olivero D, Tagliabue E, Koepsell H, Nicotra F (2014). Sodium glucose cotransporter 1 ligand BLF501 as a novel tool for management of gastrointestinal mucositis. Mol Cancer.

[39] Rapozzi V, Zorzet S, Comelli M, Mavelli I, Perissin L, Giraldi T (1998). Melatonin decreases bone marrow and lymphatic toxicity of adriamycin in mice bearing TLX5 lymphoma. Life Sci.

[40] Zordoky BN, Anwar-Mohamed A, Aboutabl ME, El-Kadi AO (2011). Acute doxorubicin toxicity differentially alters cytochrome P450 expression and arachidonic acid metabolism in rat kidney and liver. Drug Metab Dispos.

[41] Chen S, Garami M, Gardner DG (1999). Doxorubicin selectively inhibits brain versus atrial natriuretic peptide gene expression in cultured neonatal rat myocytes. Hypertension.

[42] Kim Y, Ma AG, Kitta K, Fitch SN, Ikeda T, Ihara Y, Simon AR, Evans T, Suzuki YJ (2003). Anthracycline-induced suppression of GATA-4 transcription factor: implication in the regulation of cardiac myocyte apoptosis. Mol Pharmacol.

[43] He Q, Mendez M, LaPointe MC (2002). Regulation of the human brain natriuretic peptide gene by GATA-4. Am J Physiol Endocrinol Metab.

[44] Liu X, Chua CC, Gao J, Chen Z, Landy CL, Hamdy R, Chua BH (2004). Pifithrin-alpha protects against doxorubicin-induced apoptosis and acute cardiotoxicity in mice. Am J Physiol Heart Circ Physiol.

[45] Munoz-Castaneda JR, Muntane J, Herencia C, Munoz MC, Bujalance I, Montilla P, Tunez I (2006). Ovariectomy exacerbates oxidative stress and cardiopathy induced by adriamycin. Gynecol Endocrinol.

[46] Freeland MM, Angulo J, Davis AL, Flook AM, Garcia BL, King NA, Mangibin SK, Paul KM, Prosser ME, Sata N (2012). Sex differences in improved efficacy of doxorubicin chemotherapy in Cbr1+/-mice. Anticancer Drugs.

[47] Alsaad AM, Zordoky BN, El-Sherbeni AA, El-Kadi AO (2012). Chronic doxorubicin cardiotoxicity modulates cardiac cytochrome P450-mediated arachidonic acid metabolism in rats. Drug Metab Dispos.

[48] Maayah ZH, Althurwi HN, Abdelhamid G, Lesyk G, Jurasz P, El-Kadi AO (2016). CYP1B1 inhibition attenuates doxorubicin-induced cardiotoxicity through a mid-chain HETEs-dependent mechanism. Pharmacol Res.

[49] Waxman DJ, Holloway MG (2009). Sex differences in the expression of hepatic drug metabolizing enzymes. Mol Pharmacol.

[50] Volkova M, Palmeri M, Russell KS, Russell RR (2011). Activation of the aryl hydrocarbon receptor by doxorubicin mediates cytoprotective effects in the heart. Cardiovasc Res.

[51] Anwar-mohamed A, Zordoky BN, Aboutabl ME, El-Kadi AO (2010). Alteration of cardiac cytochrome P450-mediated arachidonic acid metabolism in response to lipopolysaccharide-induced acute systemic inflammation. Pharmacol Res.

[52] Pingili AK, Kara M, Khan NS, Estes AM, Lin Z, Li W, Gonzalez FJ, Malik KU (2015). 6β-hydroxytestosterone, a cytochrome P450 1B1 metabolite of testosterone, contributes to angiotensin II-induced hypertension and its pathogenesis in male mice. Hypertension.

[53] Zordoky BN, El-Kadi AO (2010). Effect of cytochrome P450 polymorphism on arachidonic acid metabolism and their impact on cardiovascular diseases. Pharmacol Ther.

[54] Sun D, Yang YM, Jiang H, Wu H, Ojaimi C, Kaley G, Huang A (2010). Roles of CYP2C29 and RXR gamma in vascular EET synthesis of female mice. Am J Physiol Regul Integr Comp Physiol.

[55] Gonzalez FJ (2005). Role of cytochromes P450 in chemical toxicity and oxidative stress: studies with CYP2E1. Mutat Res.

[56] Lu D, Ma Y, Zhang W, Bao D, Dong W, Lian H, Huang L, Zhang L (2012). Knockdown of cytochrome P450 2E1 inhibits oxidative stress and apoptosis in the cTnT(R141W) dilated cardiomyopathy transgenic mice. Hypertension.

[57] Elkhatali S, El-Sherbeni AA, Elshenawy OH, Abdelhamid G, El-Kadi AO (2015). 19-Hydroxyeicosatetraenoic acid and isoniazid protect against angiotensin II-induced cardiac hypertrophy. Toxicol Appl Pharmacol.

[58] Qu W, Bradbury JA, Tsao CC, Maronpot R, Harry GJ, Parker CE, Davis LS, Breyer MD, Waalkes MP, Falck JR (2001). Cytochrome P450 CYP2J9, a new mouse arachidonic acid omega-1 hydroxylase predominantly expressed in brain. J Biol Chem.

[59] Muller DN, Schmidt C, Barbosa-Sicard E, Wellner M, Gross V, Hercule H, Markovic M, Honeck H, Luft FC, Schunck WH (2007). Mouse Cyp4a isoforms: enzymatic properties, gender- and strain-specific expression, and role in renal 20-hydroxyeicosatetraenoic acid formation. Biochem J.

[60] Zordoky BN, Aboutabl ME, El-Kadi AO (2008). Modulation of cytochrome P450 gene expression and arachidonic acid metabolism during isoproterenol-induced cardiac hypertrophy in rats. Drug Metab Dispos.

[61] Theken KN, Deng Y, Kannon MA, Miller TM, Poloyac SM, Lee CR (2011). Activation of the acute inflammatory response alters cytochrome P450 expression and eicosanoid metabolism. Drug Metab Dispos.

