# Supplementary material for: Sex-dependent alteration of cardiac cytochrome P450 gene expression by doxorubicin in C57Bl/6 mice
Source: Biol Sex Differ. 2017 Jan 7;8:1. doi: 10.1186/s13293-016-0124-4 (PMC5219702; doi:10.1186/s13293-016-0124-4)

**Additional file 1:** Representative RIN values. The quality of the extracted RNA was determined by measuring the RIN values using an Agilent 2100 Bioanalyzer (Santa Clara, CA).


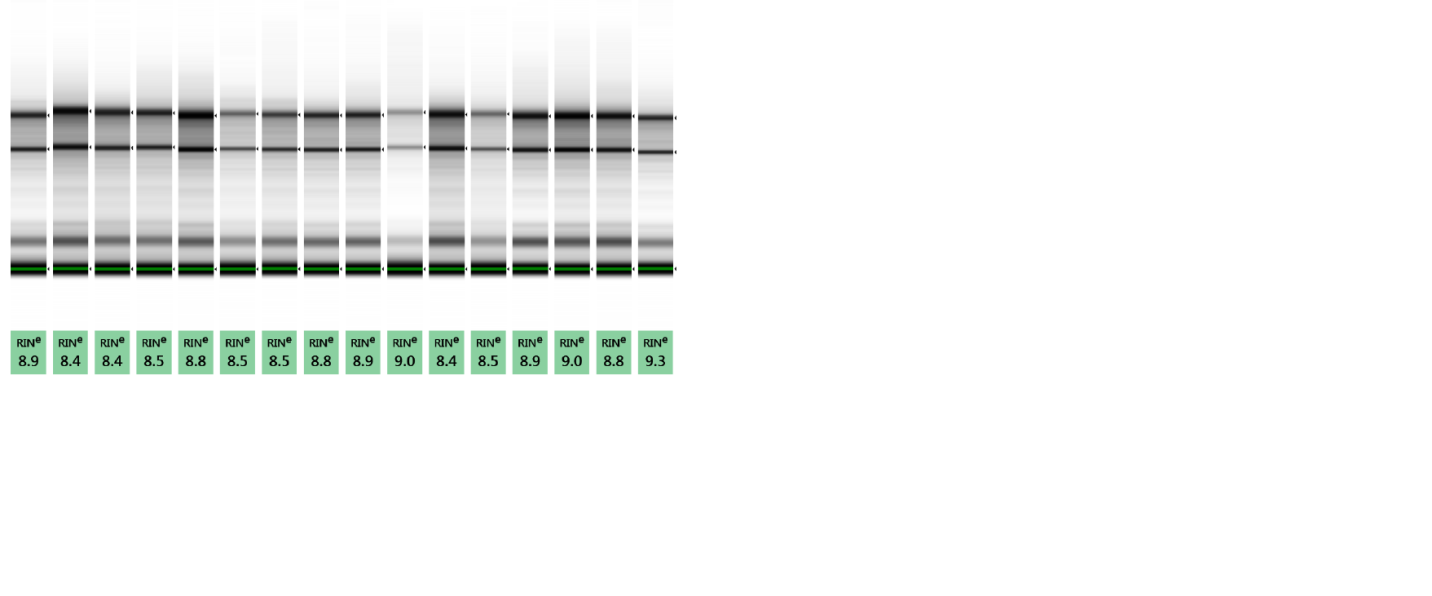

Supplement: Additional file 1: — Representative RIN values. The quality of the extracted RNA was determined by measuring the RIN values using an Agilent 2100 Bioanalyzer (Santa Clara, CA). (DOCX 111 kb) [file 13293_2016_124_MOESM1_ESM.docx]
