# Supplementary material for: Sex-dependent alteration of cardiac cytochrome P450 gene expression by doxorubicin in C57Bl/6 mice
Source: Biol Sex Differ. 2017 Jan 7;8:1. doi: 10.1186/s13293-016-0124-4 (PMC5219702; doi:10.1186/s13293-016-0124-4)
Supplement: Additional file 2: — Primer sequences used in the current study. (DOCX 15 kb) [file 13293_2016_124_MOESM2_ESM.docx]

| Gene | Forward Primer | Reverse Primer |
| --- | --- | --- |
| ANP | 5′-GGA GCC TAC GAA GAT CCA GC-3′ | 5′-TCC AAT CCT GTC AAT CCT ACC C-3′ |
| BNP | 5′-AGT CCT TCG GTC TCA AGG CA-3′ | 5′-CCG ATC CGG TCT ATC TTG TGC-3′ |
| BAX | 5′-AGCAAACTGGTGCTCAAGGC-3′ | 5′-CCACAAAGATGGTCACTGTC-3′ |
| Bcl-2 | 5′-GTGGTGGAGGAACTCTTCAG-3′ | 5′-GTTCCACAAAGGCATCCCAG-3′ |
| Cox-2 | 5′-CTG GTG CCT GGT CTG ATG ATG-3′ | 5′-GGC AAT GCG GTT CTG ATA CTG-3′ |
| Cyp1a1 | 5′-GGT TAA CCA TGA CCG GGA ACT-3′ | 5′-TGC CCA AAC CAA AGA GAG TGA-3′ |
|  | 5′-GGT TAA CCA TGA CCG GGA ACT-3′ | 5′-TGC CCA AAC CAA AGA GAG TGA-3′ |
|  | 5′-CTC TTC CCT GGA TGC CTT CAA-3′ | 5′-GGA TGT GGC CCT TCT CAA ATG-3′ |
| Cyp1b1 | 5′-AAT GAG GAG TTC GGG CGC ACA-3′ | 5′-GGC GTG TGG AAT GGT GAC AGG-3′ |
| Cyp2c29 | 5′-TGG TCC ACC CAA AAG AAA TTG A-3′ | 5′-GCA GAG AGG CAA ATC CAT TCA-3′ |
| Cyp2c44 | 5′-CTT TTC AAC GAG CGA TTC CC-3′ | 5′-TGT TTC TCC TCC TCG ATC TTG C-3′ |
| Cyp2e1 | 5′-CCC AAG TCT TTA ACC AAG TTG GC-3′ | 5′-CTT CCA TGT GGG TCC ATT ATT GA-3′ |
| Cyp2j9 | 5′-GGG AAT GTT CTA AGC CTG GAT TT-3′ | 5′-GAG TGA CTG GGC GAT TCA TAA A-3′ |
| Cyp4a10 | 5′-GTG CTG AGG TGG ACA CAT TCA T-3′ | 5′-TGT GGC CAG AGC ATA GAA GAT C-3′ |
| Beta-actin | 5′- TAT TGG CAA CGA GCG GTT CC-3′ | 5′-GGC ATA GAG GTC TTT ACG GAT GTC-3′ |
| 18S | 5′- GTA ACC CGT TGA ACC CCA TT-3′ | 5′-CCA TCC AAT CGG TAG TAG CG-3′ |

**Additional file 2:** Primer sequences used in the current study.
