# Supplementary material for: Sex-dependent alteration of cardiac cytochrome P450 gene expression by doxorubicin in C57Bl/6 mice
Source: Biol Sex Differ. 2017 Jan 7;8:1. doi: 10.1186/s13293-016-0124-4 (PMC5219702; doi:10.1186/s13293-016-0124-4)
Supplement: Additional file 3: — Kaplan-Meier survival curve for male (solid line, n = 9) or female (dashed line, n = 5) mice following a single intraperitoneal injection of 20 mg/kg doxorubicin (DOX). *p < 0.05 (DOCX 57 kb) [file 13293_2016_124_MOESM3_ESM.docx]

**Additional file 3.** Kaplan Meier survival curve for male (solid line, n = 9) or female (dashed line, n = 5) mice following a single intraperitoneal injection of 20 mg/kg doxorubicin (DOX). * P<0.05
