# Supplementary material for: Sex-dependent alteration of cardiac cytochrome P450 gene expression by doxorubicin in C57Bl/6 mice
Source: Biol Sex Differ. 2017 Jan 7;8:1. doi: 10.1186/s13293-016-0124-4 (PMC5219702; doi:10.1186/s13293-016-0124-4)
Supplement: Additional file 4: — The effect of DOX, sex, and the interaction between sex and DOX in regulating Cytochrome P450 gene expression after 24 h of administering a single intraperitoneal injection of DOX 20 mg/kg. (DOCX 15 kb) [file 13293_2016_124_MOESM4_ESM.docx]

| Gene | DOX Effect | | | | Sex Effect | | | | Interaction Effect | | | |
| --- | --- | --- | --- | --- | --- | --- | --- | --- | --- | --- | --- | --- |
|  | Effect Size (%) | F Value | P Value | Q Value | Effect Size (%) | F Value | P Value | Q Value | Effect Size (%) | F Value | P Value | Q Value |
| Cyp1a1 | 42.16 | 19.88 | 0.0001 | 0.0001* | 0.2316 | 0.1093 | 0.7435 | 0.3346 | 0.9218 | 0.4348 | 0.5153 | 0.3865 |
| Cyp1b1 | 29.84 | 36.8 | <0.0001 | <0.0001* | 40.42 | 49.85 | <0.0001 | <0.0001* | 7.042 | 8.685 | 0.0064 | 0.0200* |
| Cyp2c29 | 23.07 | 13.84 | 0.001 | 0.0012* | 28.05 | 16.82 | 0.0004 | 0.0006* | 13.94 | 8.36 | 0.0076 | 0.0200* |
| Cyp2c44 | 27.58 | 13.01 | 0.0012 | 0.0013* | 6.945 | 3.277 | 0.081 | 0.051 | 6.139 | 2.897 | 0.0998 | 0.1048 |
| Cyp2e1 | 38.37 | 22.83 | <0.0001 | <0.0001* | 10.18 | 6.059 | 0.0208 | 0.0164* | 10.18 | 6.056 | 0.0208 | 0.0364* |
| Cyp2j9 | 66.49 | 65.12 | <0.0001 | <0.0001* | 0.3392 | 0.3322 | 0.569 | 0.2994 | 4.581 | 4.487 | 0.0432 | 0.0567 |
| Cyp4a10 | 56.9 | 52.04 | <0.0001 | <0.0001* | 15.29 | 13.99 | 0.0009 | 0.0009* | 0.75 | 0.69 | 0.4145 | 0.3627 |

**Additional file 4: The effect of DOX, sex, and the interaction between sex and DOX in regulating Cytochrome P450 gene expression after 24 hours of administering a single intraperitoneal injection of DOX 20 mg/kg.** Comparisons among different sex and treatment groups were done by 2-way ANOVA (DFn = 1 and DFd = 28). To correct for multiple comparisons, a false discovery rate of 5% was applied for p values of DOX effect, sex effect, and interaction effect on Cytochrome P450 gene expressions by the Two-stage linear step-up procedure of Benjamini, Krieger and Yekutieli. * q<0.05 denotes discoveries.
