# Supplementary material for: Sex-dependent alteration of cardiac cytochrome P450 gene expression by doxorubicin in C57Bl/6 mice
Source: Biol Sex Differ. 2017 Jan 7;8:1. doi: 10.1186/s13293-016-0124-4 (PMC5219702; doi:10.1186/s13293-016-0124-4)
Supplement: Additional file 5: — The effect of DOX, sex, and the interaction between sex and DOX in regulating Cytochrome P450 gene expression after 6 days of administering a single intraperitoneal injection of DOX 20 mg/kg. (DOCX 15 kb) [file 13293_2016_124_MOESM5_ESM.docx]

**Additional file 5: The effect of DOX, sex, and the interaction between sex and DOX in regulating Cytochrome P450 gene expression after 6 days of administering a single intraperitoneal injection of DOX 20 mg/kg:** Comparisons among different sex and treatment groups were done by 2-way ANOVA (DFn = 1 and DFd = 16). To correct for multiple comparisons, a false discovery rate of 5% was applied for p values of DOX effect, sex effect, and interaction effect on Cytochrome P450 gene expressions by the Two-stage linear step-up procedure of Benjamini, Krieger and Yekutieli. * q<0.05 denotes discoveries.

| Gene | DOX Effect | | | | Sex Effect | | | | Interaction Effect | | | |
| --- | --- | --- | --- | --- | --- | --- | --- | --- | --- | --- | --- | --- |
|  | Effect Size (%) | F Value | P Value | Q value | Effect Size (%) | F Value | P Value | Q Value | Effect Size (%) | F Value | P Value | Q Value |
| Cyp1a1 | 1.925 | 0.5622 | 0.4658 | 0.4935 | 46.01 | 13.44 | 0.0025 | 0.0064* | 15.17 | 4.432 | 0.0538 | 0.1977 |
| Cyp1b1 | 43.15 | 22.91 | 0.0002 | 0.0013* | 20.44 | 10.85 | 0.0046 | 0.0064* | 15.44 | 8.196 | 0.0113 | 0.0831 |
| Cyp2c29 | 0.0962 | 0.0019 | 0.9556 | 0.86 | 24.74 | 4.98 | 0.0412 | 0.0433* | 0.0159 | 0.0032 | 0.9556 | 1.0000 |
| Cyp2c44 | 17.75 | 5.863 | 0.0286 | 0.08 | 34.68 | 11.46 | 0.0041 | 0.0064* | 2.312 | 0.7639 | 0.3959 | 0.9700 |
| Cyp2e1 | 25.47 | 5.17 | 0.0381 | 0.08 | 0.0884 | 0.018 | 0.8952 | 0.5371 | 0.773 | 0.1569 | 0.6976 | 1.0000 |
| Cyp2j9 | 7.966 | 1.615 | 0.2232 | 0.2812 | 16.61 | 3.366 | 0.0865 | 0.0727 | 0.003 | 0.0006 | 0.9806 | 1.0000 |
| Cyp4a10 | 18.28 | 4.101 | 0.0599 | 0.0943 | 6.25 | 1.402 | 0.2537 | 0.1776 | 1.147 | 0.2572 | 0.619 | 1.0000 |
